# Development and validation of a lectin-based assay for detection of IgG Fc glycosylation as a biomarker in lupus nephritis

**DOI:** 10.1136/lupus-2026-002059

**Published:** 2026-07-02

**Authors:** Rohit Upadhyay, Juan Gao, Alexia M Michelle Orellana Enamorado, Scott E Wenderfer, George C Tsokos, Rhea Bhargava

**Affiliations:** 1 Tulane University School of Medicine, New Orleans, Louisiana, USA; 2 Pediatrics, Baylor College of Medicine, Houston, Texas, USA; 3 Harvard Medical School, Boston, Massachusetts, USA

**Keywords:** Lupus Erythematosus, Systemic, Lupus Nephritis, Antibodies

## Abstract

**Background:**

Lupus nephritis (LN) is a devastating complication of systemic lupus erythematosus (SLE). Aberrant IgG glycosylation drives podocyte injury and is a robust marker for LN. The primary method for detecting glycosylation is limited to mass spectrometry, but it is not suitable for clinical applications due to its complexity and high cost.

**Methods:**

We developed and optimised a lectin-based enzyme-linked assay to detect IgG glycan residues. Lectins were selected based on the glycan structures detected in a SLE cohort and were differentially expressed in SLE with and without nephritis. 96-well plates were coated with protein L. Blocking solutions for each lectin were selected from 5% bovine serum albumin (BSA), deglycosylated BSA (deBSA) and carbo-free blocking solution. IgG standards range was optimised starting from 0 to 20 µg/mL. Fucose was detected by *Aleuria aurantia* lectin (AAL), galactose was detected by *Erythrina crista-galli* lectin (ECL) and sialic acid was detected by *Sambucus nigra* lectin (SNA). An SLE cohort was used to test the ability of these novel assays to differentiate patients with LN from non-renal SLE.

**Results:**

The selected lectins produced reproducible standard curves with strong linearity. ELISA assays performed using IgG from a healthy donor, and a patient with LN revealed glycan differences that were consistent with mass spectrometry data and within range of the standard curves. While the AAL (fucose) binding did not differ among different SLE subgroups, ECL (galactose) binding showed significant differences in patients with active LN compared with those with active non-renal SLE and quiescent disease. SNA (sialic acid) binding also distinguished patients with active LN from those with quiescent SLE; however, it did not discriminate between renal and non-renal SLE.

**Conclusions:**

The developed lectin-ELISA assay can detect glycosylation characteristics of IgG in a more cost-effective, clinically applicable manner compared with mass spectrometry. Our validation data suggest the potential of ECL binding assays to help diagnose and monitor patients with LN.

**WHAT IS ALREADY KNOWN ON THIS TOPIC**

- 40%–60% of systemic lupus erythematosus (SLE) patients develop lupus nephritis (LN) and identifying these individuals remains a significant clinical challenge with profound implications for morbidity, mortality and healthcare burden. Currently LN is diagnosed by invasive kidney biopsy, which is difficult to repeat to monitor disease activity. Prior studies have shown that IgG glycosylation is a robust biomarker of differentiating kidney involvement in SLE. However, current methods such as mass spectrometry to measure glycans are costly and impractical for routine clinical use, highlighting the need for simpler noninvasive diagnostic tools.

**WHAT THIS STUDY ADDS**

- This study proposes a lectin-based ELISA assay that reliably detects IgG glycosylation patterns. Furthermore, this study demonstrates that ECL binding can distinguish patients with active LN from other SLE subgroups.

**HOW THIS STUDY MIGHT AFFECT RESEARCH, PRACTICE OR POLICY**

- The findings suggest a feasible, cost-effective alternative for potential use in diagnosing and monitoring LN. This could improve accessibility of biomarker testing and guide future research into scalable diagnostic assays.

## Introduction

*Systemic lupus erythematosus (SLE*) is an autoimmune disease characterised by autoantibody production and immune complex deposition, leading to multi-organ inflammation. Among its manifestations, *lupus Nephritis (LN*) is particularly devastating, affecting up to 60% of SLE patients and serving as a major determinant of morbidity and mortality.^1 2^ Non-invasive serologic monitoring of LN activity remains a challenge, as current biomarkers such as antibodies to dsDNA and complement levels lack sensitivity and specificity.^3^

Aberrant *IgG glycosylation*, especially reduced galactosylation and sialylation, has emerged as a promising biomarker for autoimmune disease activity, including LN.^4 5^ The IgG Fc region carries a conserved N-linked glycan at Asn297, whose composition modulates Fc receptor binding and effector functions.^6^ Hypogalactosylated and hyposialylated IgG promote pro-inflammatory effector activity, whereas sialylated IgG exhibits anti-inflammatory properties.^7 8^

Mass spectrometry-based glycomics analysis has confirmed disease-specific glycan alterations in SLE and LN.^9 10^ However, Liquid Chromatograph Triple Quadrupole Mass Spectrometer (LC-MS/MS) assays are resource-intensive, technically complex and ill-suited for routine diagnostic laboratories. Lectin-based approaches, exploiting the carbohydrate-binding specificity of plant-derived lectins, should offer a simpler, low-cost and high-throughput alternative for glycan profiling.^11^

Here, we report the development and optimisation of a lectin-based enzyme-linked assay to detect key IgG glycan moieties (fucose, galactose and sialic acid). This work represents a step towards a clinically applicable diagnostic platform for early LN detection and disease monitoring.

## Materials and methods

### Plate coating and blocking optimisation

96-well plates were coated with protein L (which binds kappa light chains of IgG independent of subclass) to capture total IgG. Blocking reagents tested included 5% bovine serum albumin (BSA), deglycosylated BSA (deBSA) and a carbohydrate-free blocking solution (Vector Laboratories, VBS).

### Lectin binding

Each lectin was optimised for signal-to-noise ratio and linearity:

- *Aleuria aurantia lectin (AAL):* specific for *fucose.*
- *Erythrina cristagalli lectin (ECL):* specific for *galactose.*
- *Sambucus nigra lectin (SNA):* specific for *α2,6-linked sialic acid.*

IgG standards were serially diluted (0–20 µg/mL) to establish calibration curves. Bound lectins were detected using biotin–streptavidin–horseradish peroxidase (HRP) and tetramethylbenzidine (TMB) substrate, read at 450 nm.

### Clinical samples

Plasma IgG was isolated from four groups of patients: (1) active LN, (2) active SLE without renal involvement, (3) quiescent SLE and (4) LN patients after 6 months of immunosuppressive therapy as described in a previous publication.^9^ Sample and paired data collection was approved by the institutional review board at Baylor College of Medicine. Informed assent and consent were obtained from the patients and caregivers before enrolling in the study, in accordance with principles of the Declaration of Helsinki.

### ELISA to detect glycans on IgG with lectins

#### Reagents

- Protein L (Thermo Fisher, Cat# 21189).
- IgG standard (QA Bio, Cat# GCP-1GG-100U).
- ECL biotinylated (Vector Lab, B-1145-5).
- SNA biotinylated (Vector Lab, Cat# B-1305-2).
- Streptavidin-HRP (Thermo Fisher, Cat# N100).
- HRP substrate (Vector Lab, Cat# TMB SK-4400).
- Carbo-free blocking solution (VBS) (Vector Lab, Cat# SP-5040-125).
- PBS (Thermo Fisher, Cat# 10010023).

### Preparation of IgG standards (0, 5, 10, 20 µg/mL)

**Table:** 

Working solution Concentration (µg/mL)	Working solution Volume (µL)	Stock solution (µL)	H2O (µL)
0	1000	0	1000
5	1000	25	975
10	1000	50	950
20	1000	100	900

### Protocol

**Figure 1:**
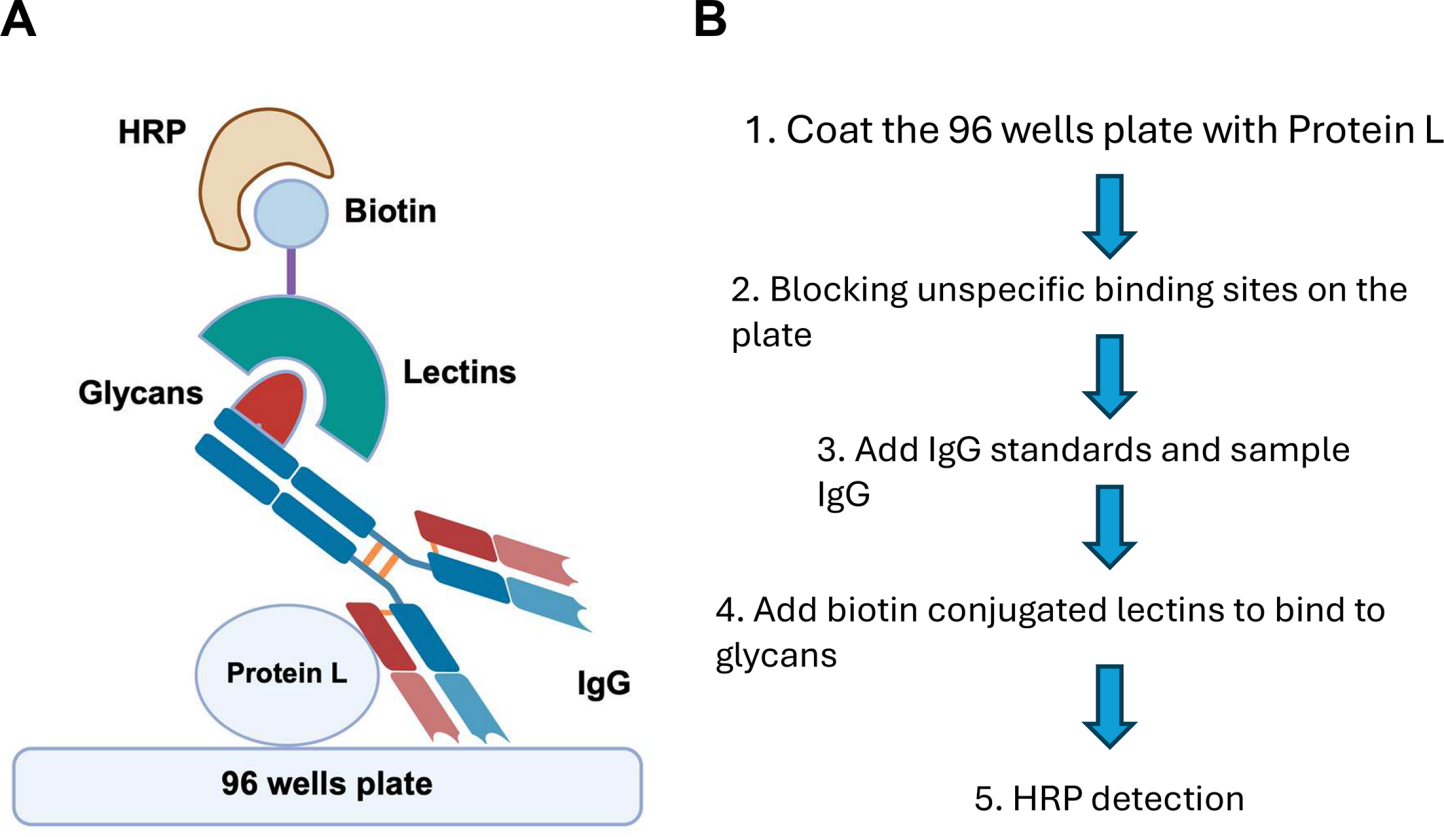
(A) Schematic depicting the order of reagents and (B) flow chart for lectin-ELISA assay.

Below are the steps used to perform Lectin-ELISA (figure 1A and B):

1. Plate coating with Protein L (Stock solution 50 µg/mL).
2. Dilute Protein L in coating buffer (Carbonate bicarbonate buffer pH 9.4) to 5 µg/mL working concentration.
3. Add 100 µl Protein L working solution to each well of the 96-well plate.
4. Incubate overnight at 4 °C.
5. Wash the plate 3 times with 300 µL Phosphate-Buffered Saline with Tween 20 (PBST) phosphate buffer solution.
6. Block the plate with VBS for 1 hour at room temperature.
7. Wash the plate one time.
8. Add IgG standards or sample, 100 µL/well, incubate overnight at 4 °C. IgG standards can be diluted in PBST.
9. Add 100 µL of sample to each well. (1 µg/well for ECL, 0.5 µg/well for AAL and SNA).
10. Wash the plate with PBST three times.
11. Add lectin, Room temperature (RT), 1 hour.
  - ECL, working solution 5 µg/mL, 100 µL per well.
  - SNA, 1 µg/mL, 100 µL per well.
  - AAL, 2 µg/mL, 100 µL per well.
12. Wash the plate three times.
13. Add streptavidin-HRP, 1:5000, RT, 1 hour.
14. Wash the plate three times.
15. Add HRP substrate and TMB peroxide substrate in 5 mL water, and add two drops Reagent 1, three drops Reagent 2 and two drops of Reagent 4. 100 µL/well; incubate for 30 min, RT.
16. Add sulfuric acid 1N (50 µL/well) to stop the reaction, then read the plate at 450 nm.

#### IgG isolation

IgG was isolated from plasma using a Melon Gel IgG Spin Purification Kit (Thermo Scientific; catalogue no. 45206), following manufacturer’s instructions. IgG was subsequently added to the ELISA plates.

#### Patient and public involvement

It was not appropriate or possible to involve patients or the public in the design, conduct, reporting or dissemination plans of our research.

## Results

### Designing lectin-ELISA assay

Plate wells were coated with Protein L to capture IgG through its light chain, thereby preserving glycan accessibility on the Fc region for subsequent lectin binding. Although Protein L can also bind IgM, IgA and IgE, this was not expected to affect assay performance because purified IgG was used. This approach ensured consistent antibody capture while maintaining the integrity of Fc-associated glycans for lectin-based detection. The overall assay design involved four sequential stages: (1) Protein L coating and plate preparation; (2) IgG loading from standards or clinical samples; (3) lectin incubation using AAL, ECL or SNA to detect fucose, galactose and sialic acid respectively; and (4) enzymatic signal development using streptavidin-HRP and TMB substrate. Figure 1A shows a schematic of the lectin-based assay, and figure 1B illustrates the stepwise workflow, which was optimised to maintain glycan integrity, reduce background signal and support reproducible lectin binding.

### Blocking optimisation

**Figure 2:**
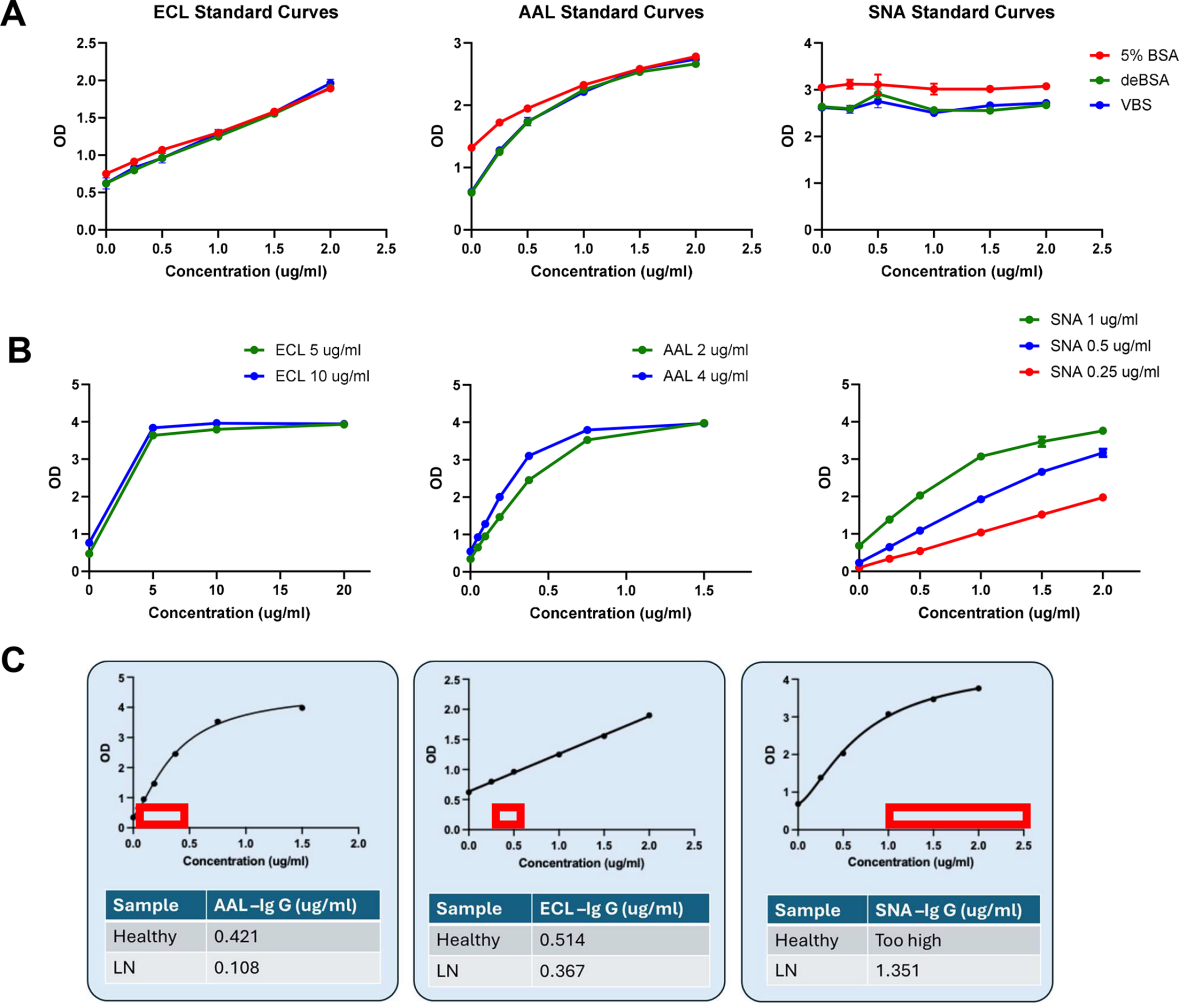
Optimising the lectin-ELISA. (**A**) Selection of blocking solution. 5% BSA; deglycosylated BSA, deBSA, 5% BSA with 50 mM sodium periodate in 50 mM sodium acetate (pH 4.0), dialysis in Tris-Buffered Saline (TBS), 2 hour Room temperature (RT) and 4 °C overnight; VBS: carbo-free blocking solution from Vector Lab. (**B**) Selection of lectins and optimisation of optimal concentrations. Several lectin concentrations were used to develop standard curves and optimise the working conditions for the lectin-ELISA. (**C**) Assay validation in samples from humans. Human IgG from a healthy donor and from a SLE patient were used to test the concentration ranges of the lectin-ELISAs. The red boxes depict the lowest and highest concentration noted in these samples. BSA, bovine serum albumin.

Blocking studies showed that both AAL and SNA exhibited cross-reactivity when 5% BSA was used, indicating interference from glycans present in standard BSA. In contrast, deglycosylated BSA (deBSA) and the VBS effectively eliminated non-specific binding for AAL and SNA. All blocking agents, including 5% BSA, were suitable for ECL, which did not display glycan-dependent background signals. Based on these results, deBSA and VBS were identified as the most reliable blocking solutions for lectin-based detection in this assay (figure 2A).

### Lectin selection and optimisation of working concentrations

AAL (specific for fucose), ECL (specific for galactose) and SNA (specific for α2,6-linked sialic acid) were evaluated for assay development. The optimised standard curve ranges for IgG-lectin binding were as follows: AAL, (0–4 µg/mL), ECL (0–20 µg/mL) and SNA (0–2 µg/mL).

To achieve robust sigmoid standard curves, the optimal working concentrations for the lectins were: ECL at 5 µg/mL and 10 µg/mL, AAL at 2 µg/mL and 4 µg/mL and SNA at 1 µg/mL. The assay demonstrated excellent reproducibility, with linear correlation coefficients (R² > 0.98) (figure 2B).

### Assay validation using samples from a healthy donor and an LN patient

To evaluate assay performance in human biological samples, we tested IgG isolated from a healthy donor and a patient with LN. Standard curves were generated by loading 0, 5, 10 and 20 µg/mL IgG (100 µL/well) and incubating plates overnight at 4 °C. IgG stock (200 µg/mL) was prepared by reconstituting 100 µg lyophilised IgG in 0.5 mL ultrapure water. Sample working solutions were prepared by dilution in assay buffer (healthy donor, 3.99 µg/µL stock; LN, 1.69 µg/µL stock), yielding final assay concentration of 0.01 µg/µL (100 µL/well).

Across all three lectins, signals from the standard curves were consistent with the expected dynamic ranges for AAL, ECL and SNA (figure 2C). AAL binding to IgG was noted in both SLE and healthy donors; however, it was markedly reduced in the SLE sample compared with the healthy donor (0.108 vs 0.421 µg/mL AAL-IgG equivalents). ECL binding was also lower in the SLE sample (0.367 vs 0.514 µg/mL). SNA showed substantially lower binding in the SLE sample (1.351 µg/mL), compared with the healthy control.

Together, these results suggest that the developed optimised lectin-based assays can detect glycosylation differences in clinical samples and may be suitable for detecting SLE-associated IgG glycan alterations (figure 2C).

### Glycosylation profiles in clinical samples and assay validation in SLE patient cohorts

We studied 30 children with a diagnosis of SLE who enrolled into the study from a single centre in Houston, Texas, between 2015 and 2021. Inclusion criteria in the cohort included age 2 to 18 years and having at least 4 of 11 classification criteria from the 1997 revised American College of Rheumatology system for SLE. Clinical and histopathologic information was extracted from patient records. Systemic Lupus Erythematosus Disease Activity Index (SLEDAI) 2000 scores were used to assess disease activity and severity, and renal SLEDAI (rSLEDAI) scores were calculated using the four urinary metrics of the SLEDAI. We also analysed 10 paired samples from children with LN, collected before and 6 months after initiation of immunosuppressive treatment for LN. LN was diagnosed with a kidney biopsy read by a renal pathologist. Immunosuppressive therapy consisted of hydroxychloroquine and glucocorticoids along with various combinations of mycophenolate, cyclophosphamide, anti-CD20 and intravenous IgG. Samples that were obtained within 90 days of intravenous IgG administration were excluded from this analysis. Demographics for this cohort are represented in table 1.

**Table 1:** Demographics of the individuals in the SLE cohort

Baseline characteristic	SLE without LN	LN	Quiescent SLE	LN after treatment
Female, n (%)	9 (90)	8 (80)	7 (70)	8 (80)
Male, n (%)	1 (10)	2 (20)	3 (30)	2 (20)
Age (years)	10.51–18.06	11.28–16.57	13.01–17.61	11.78–17.07
SLEDAI, range	9–17	10–33	0–3	4–25
Renal SLEDAI, range	0	8–16	0	0–16
UPCR, range (mg/mg)	0.06–0.26	0.3–15	0.02–0.11	0.08–5.8
Serum Cr, range (mg/dL)	0.4–0.7	0.4–2.0	0.5–0.7	0.4–1.4
eGFR, range (mL/1.73 m²/min)	82–137	31–186	73–124	46–132
Anti-dsDNA, range (IU)	0–1280	0–2560	0–1280	0–1280
C3ᵃ, range (mg/dL)	14–126	5–51	89–151	5–118

dsDNA, Double-stranded DNA; eGFR, estimated glomerular filtration rate and; LN, lupus nephritis; SLE, systemic lupus erythematosus; SLEDAI, Systemic Lupus Erythematosus Disease Activity Index.

**Figure 3:**
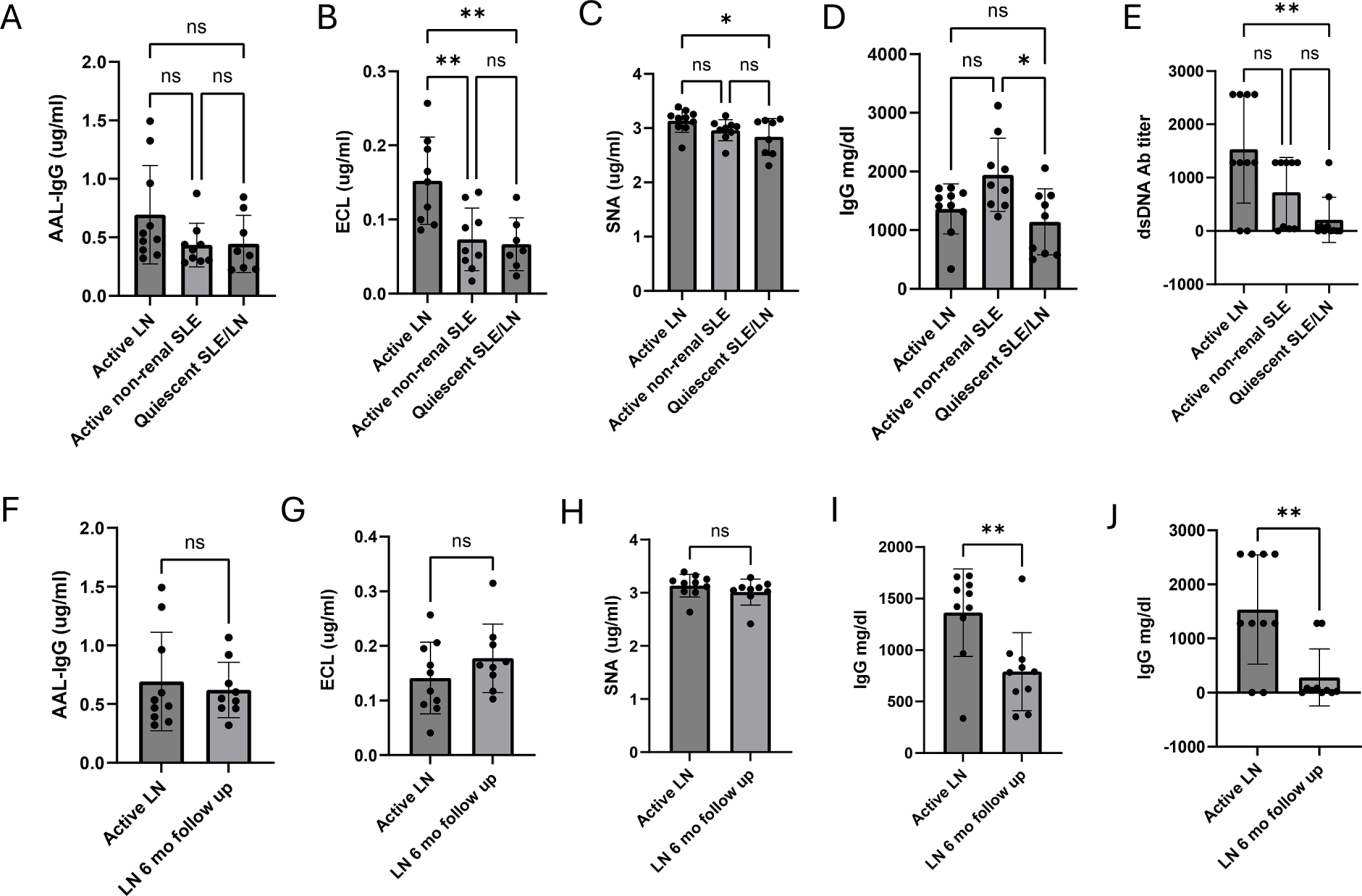
Lectin-ELISA to monitor LN activity in a cohort of individuals with SLE. (**A**) AAL ELISA did not show differences between Active LN, non-renal SLE and quiescent SLE. (**B**) ECL lectin ELISA was able to differentiate between active LN, active non-renal SLE and quiescent SLE. (**C**) No differences were noted in SNA lectin binding assays except between active LN and quiescent SLE. (**D–E**). Total IgG levels and dsDNA ab titers did not show differences between active LN and active non-renal SLE. (**F–H**). No differences were noted in AAL, ECL or SNA binding between 6-month paired samples pre- and post-induction therapy in those with LN. (**I–J**). IgG and dsDNA ab titers decreased after induction therapy in LN. Data are presented as means±SEMs (n=10). Statistical differences were determined using one-way analysis of variance or two-tailed t-test. *p<0.05, ** p<0.01, ns, nonsignificant. AAL, *Aleuria aurantia* lectin; ECL, *Erythrina crista-galli* lectin; LN, lupus nephritis; SLE, systemic lupus erythematosus; SNA, *Sambucus nigra* lectin.

Using the optimised lectin assay, we quantified IgG glycosylation in samples from patients with active LN, active SLE, quiescent SLE and treated LN. Consistent with published LC-MS/MS analyses,^9^ no significant differences in fucosylation (figure 3A) were observed across the four clinical groups, indicating that fucosylation remains unchanged across SLE disease states.

ECL binding assay, which detects galactose, was able to differentiate active LN from active non-renal SLE (figure 3B) and those with quiescent SLE (figure 3B). No differences in ECL binding were noted between non-renal SLE and quiescent SLE.

Next, we tested SNA binding in this cohort. No differences were detected in the group with active LN when compared with non-renal SLE; however, significant differences were noted between active LN and quiescent SLE (figure 3C). SNA binding was similar in active non-renal SLE and quiescent SLE. Next, we compared total IgG levels and dsDNA antibody titers between these groups. Both total IgG and dsDNA antibody levels were unable to differentiate between active LN and active non-renal SLE (figure 3D and E). We also found no statistically significant differences in samples pre and post induction therapy in ECL or SNA binding (figure 3F and H). Both total IgG and dsDNA antibody levels decreased post induction therapy in LN (figure 3I and J).

ECL binds strongly to non-sialylated, terminal (LacNAc) sequences, particularly when presented on poly-N-acetyllactosamine chains.^12^ While it recognises galactose and N-acetylgalactosamine, sialylation generally inhibits ECL binding, making it an ideal tool for detecting desialylated or ‘exposed’ glycans.^13^ In a previous study, we detected a glycan structure which was core fucosylated, non-sialylated, galactosylated glycan capable of differentiating active LN from active non-renal SLE. The results in this study suggest our ECL binding may be able to detect these glycan structures as demonstrated by these figures along with monitoring LN activity in SLE.

**Figure 4:**
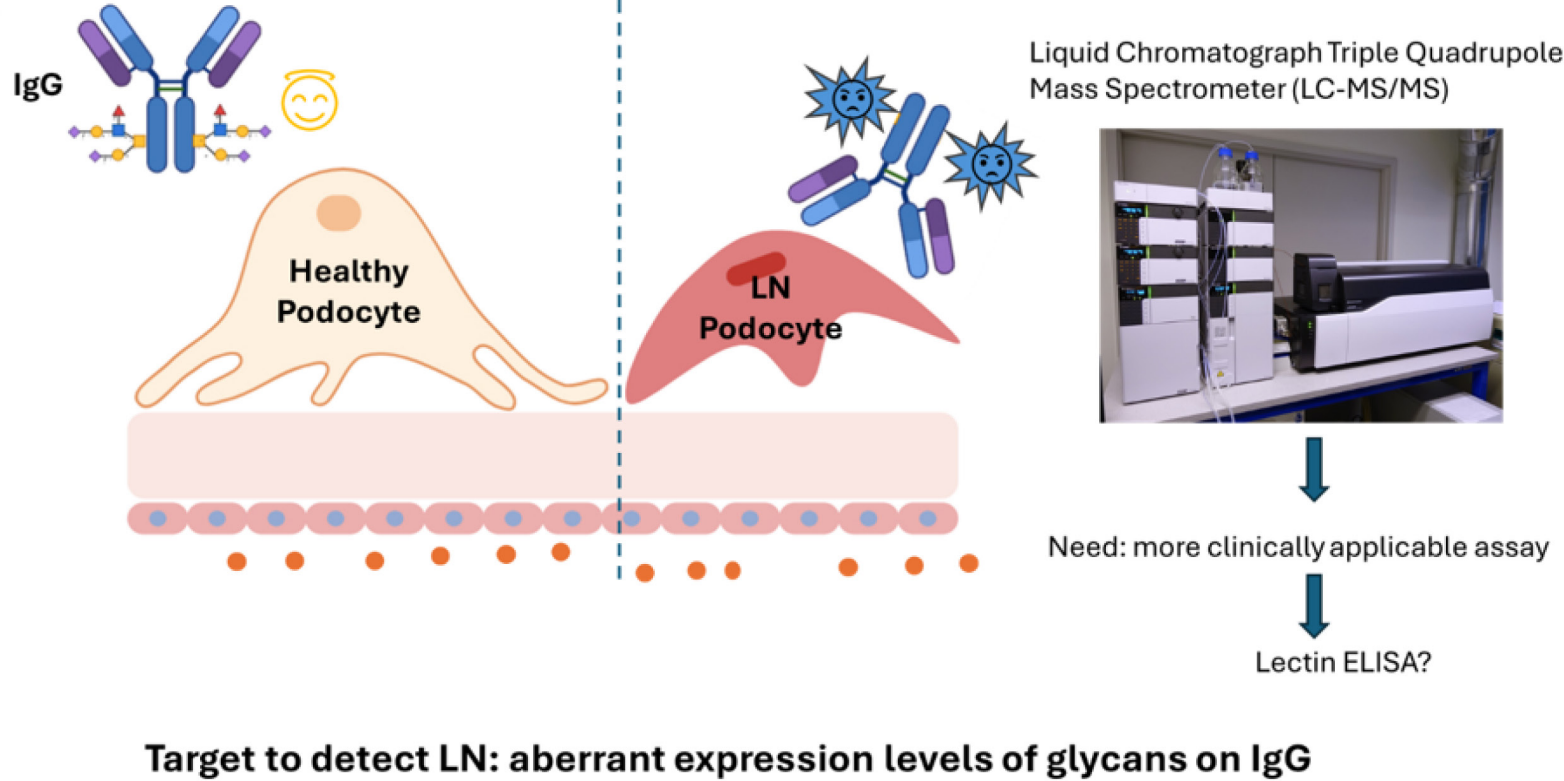
Aberrantly glycosylated IgG is present in Lupus Nephritis and damages podocytes. Detection of glycosylation patterns requires the cumbersome use of Liquid Chromatograph Triple Quadrupole Mass Spectrometer (LC-MS/MS). The proposed lectin-based detection of glycan residues is easy to use and clinically applicable.

Overall, these data demonstrate that the lectin-based assay reliably captures group-level glycan patterns for fucose, galactose and sialic acid residues (figure 4). Furthermore, ECL binding in combination with SNA binding to IgG can distinguish active LN from active non-renal SLE and quiescent SLE. Preliminary comparisons with LC-MS/MS support the assay’s accuracy and suitability for profiling IgG glycosylation in diverse SLE phenotypes, particularly for differentiating active LN from non-renal SLE (figures3  4).

## Discussion

This study demonstrates the feasibility of a lectin-based platform to profile IgG glycosylation in patients with SLE and LN. Our results confirm that specific lectin–carbohydrate interactions can reliably measure major glycan moieties relevant to immune regulation.

The pathophysiological relevance of IgG glycosylation in LN has been well-documented. Hypogalactosylated IgG correlates with complement activation and disease activity,^4 14^ while desialylation enhances proinflammatory FcγRIII binding.^7^ Elevated afucosylated IgG is known to potentiate antibody-dependent cellular cytotoxicity in other autoimmune and infectious diseases,^15^ though its role in LN remains less clear. We have demonstrated previously that IgG associated glycans can be robust biomarkers for SLE and aberrantly glycosylated IgG characterises LN.^9 16^ However, these studies were performed using mass spectrometry detection methods.

By replacing mass spectrometry with lectin-based detection, our assay substantially reduces assay time and cost, offering potential clinical utility. Importantly, the approach maintains fidelity with LC-MS/MS data, underscoring its analytical validity. The current findings also highlight that fucosylation may not be a dynamic biomarker of LN activity, whereas future iterations could incorporate galactose and sialic acid quantification to refine disease stratification. ECL binding in particular may have utility in diagnosing and monitoring renal involvement in SLE. Furthermore, these assays were more effective at differentiating active LN from active non-renal SLE.

Emerging literature supports integrating glycan-based biomarkers with standard serological and proteomic markers for early LN prediction.^5^ Thus, a standardised, high-throughput lectin assay could complement traditional immunological assays, offering a non-invasive and quantitative readout of LN and kidney disease.

Quantification of IgG glycans, including fucose, sialic acid and galactose, currently relies on LC-MS/MS-based methods, which limits their routine use in clinical settings. The present assay requires a turnaround time of approximately 48 hours, indicating that further optimisation will be necessary to reduce processing time and enhance clinical practicality. In addition, expanding the analysis to include glycan species beyond the three evaluated in this study may improve the diagnostic resolution of the assay. Finally, broader validation is required to support clinical implementation, including longitudinal studies of samples from patients with active LN and the assessment of glycan changes in response to treatment. Since the dsDNA antibody ELISA showed trends similar to the lectin IgG assay when comparing active LN and inactive disease, although without attaining statistical significance, both assays need to be validated using larger patient cohorts.

In summary, the current lectin assay represents a plausible tool for detecting fucose, sialic acid and galactose residues on IgG derived from human samples. This work establishes a proof of concept for a lectin-based IgG glycosylation assay that is simple, reproducible and cost-effective. Such a platform has the potential to be adapted for routine clinical testing, enabling earlier detection of glycosylation changes associated with the onset of LN or responses to treatment. Notably, the ECL lectin assay demonstrated strong potential in identifying active renal involvement in patients with SLE. Future studies will aim to incorporate additional lectins and multiplexed detection strategies to capture a broader range of glycan diversity and further enhance diagnostic capability.

## Data Availability

Data sharing not applicable as no datasets generated and/or analysed for this study.

## References

[1] Anders HJ, Saxena R, Zhao MH (2020). Lupus nephritis. Nat Rev Dis Primers.

[2] Almaani S, Meara A, Rovin BH (2017). Update on Lupus Nephritis. Clin J Am Soc Nephrol.

[3] Parikh SV, Almaani S, Brodsky S (2020). Update on Lupus Nephritis: Core Curriculum 2020. Am J Kidney Dis.

[4] Zhou X, Motta F, Selmi C (2021). Antibody glycosylation in autoimmune diseases. Autoimmun Rev.

[5] Huang L, Shi J, Li H (2025). Integrated multi-omics analysis reveals diagnostic biomarkers and therapeutic targets for systemic lupus erythematosus. Medicine (Baltimore).

[6] Arnold JN, Wormald MR, Sim RB (2007). The impact of glycosylation on the biological function and structure of human immunoglobulins. Annu Rev Immunol.

[7] Kaneko Y, Nimmerjahn F, Ravetch JV (2006). Anti-inflammatory activity of immunoglobulin G resulting from Fc sialylation. Science.

[8] Karsten CM, Pandey MK, Figge J (2012). Anti-inflammatory activity of IgG1 mediated by Fc galactosylation and association of FcγRIIB and dectin-1. Nat Med.

[9] Bhargava R, Upadhyay R, Zhao C (2025). Aberrant Glycosylation of IgG in Children With Active Lupus Nephritis Alters Podocyte Metabolism and Causes Podocyte Injury. *Arthritis Rheumatol*.

[10] Bondt A, Hafkenscheid L, Falck D (2018). ACPA IgG galactosylation associates with disease activity in pregnant patients with rheumatoid arthritis. Ann Rheum Dis.

[11] Yamada M, Hirabayashi J, Nishihara S, Angata K, Aoki-Kinoshita KF (2021). Glycoscience protocols (GlycoPODv2).

[12] Bojar D, Meche L, Meng G (2022). A Useful Guide to Lectin Binding: Machine-Learning Directed Annotation of 57 Unique Lectin Specificities. ACS Chem Biol.

[13] Cao X, Wang S, Gadi MR (2022). Correction: Systematic synthesis of bisected *N*-glycans and unique recognitions by glycan-binding proteins. Chem Sci.

[14] Gornik O, Pavić T, Lauc G (2012). Alternative glycosylation modulates function of IgG and other proteins - implications on evolution and disease. Biochim Biophys Acta.

[15] Oosterhoff JJ, Larsen MD, van der Schoot CE (2022). Afucosylated IgG responses in humans - structural clues to the regulation of humoral immunity. Trends Immunol.

[16] Bhargava R, Lehoux S, Maeda K (2021). Aberrantly glycosylated IgG elicits pathogenic signaling in podocytes and signifies lupus nephritis. JCI Insight.

